# Resilience in Family caregivers of Tunisian patients with psychiatric disorders

**DOI:** 10.1192/j.eurpsy.2022.1611

**Published:** 2022-09-01

**Authors:** K. Safa, N. Charfi, M. Marwa, I. Gassara, R. Feki, N. Smaoui, S. Omri, M. Maalej, L. Zouari, J. Ben Thabet, M. Maalej

**Affiliations:** Hedi Chaker University hospital, Psychiatry C, sfax, Tunisia

**Keywords:** resilience-caregivers-psychiatric disorders-patients

## Abstract

**Introduction:**

Resilience has been described as an individual’s ability to adjust or adapt to significant adverse or traumatic circumstances.

**Objectives:**

The aims of this study were to assess the resilience of caregivers of patients with mental disorders and to identify its associated factors.

**Methods:**

We conducted a descriptive and analytical cross-sectional study among caregivers of patients followed in the outpatient psychiatry department at the university hospital of Sfax(Tunisia), during septembre 2021. Resilience was assessed using Connor–Davidson Resilience Scale(CD‑RISC).The total scores range from zero to 100. The cut-off scores for this questionnaire is 50, with higher score indicating higher resilience.

**Results:**

Our sample included 34 family caregivers. The mean age was 47.47 years (SD=12.4 years)and the sex ratio (M/F) was 1.42. They were the parents of patients in 35.3% of cases. The mean duration of providing care to patients was 8.62 years. The mean resilience score of caregivers was 42.85 and 26.5% of them were resilients. The Caregivers with low socioeconomic level (p=0.004), a history of chronic illness (p=0.0001), a long duration of providing care (p=0.001), a stressful events (0.002) and those victims of agressive behaviors committed by patients (p=0.0001) were more likely to have a low resilience score.

**Conclusions:**

Our results stated that three out four cargivers have low level of resilience. Interventions targeting stress related to social events and burden of care, and violence committed by patients in their family environment should be integrated to increase the caregivers’resilience.

**Disclosure:**

No significant relationships.

